# P-841. Empiric Antimicrobial Treatment of Sepsis in Canadian Emergency Rooms: Analysis of Pathogens Causing Bacteremia and Their Susceptibility Rates

**DOI:** 10.1093/ofid/ofae631.1033

**Published:** 2025-01-29

**Authors:** Jeremy Li, Melanie R Baxter, Andrew Walkty, James Karlowsky, George Zhanel

**Affiliations:** University of Manitoba, Winnipeg, Manitoba, Canada; University of Manitoba, Winnipeg, Manitoba, Canada; University of Manitoba, Winnipeg, Manitoba, Canada; University of Manitoba, Winnipeg, Manitoba, Canada; University of Manitoba, Winnipeg, Manitoba, Canada

## Abstract

**Background:**

An alarming increase in the frequency of ESBL-producing organisms has been reported in Canada, raising concerns about whether empiric regimens used for the treatment of sepsis remain sufficient to cover ER patients with potentially undiagnosed bloodstream infections. Using data from the CANWARD study, we report on the epidemiology of bloodstream infections in Canadian emergency rooms and estimate the susceptibility rate of these pathogens to empiric regimens commonly used for the treatment of sepsis.

**Figure:**
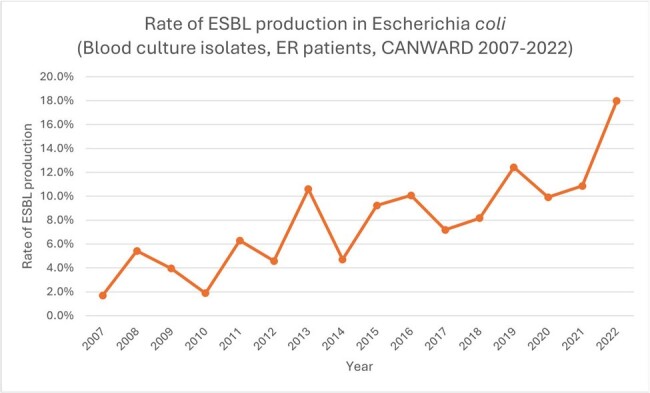

Rate of ESBL production in blood culture isolates of Escherichia coli (ER patients, CANWARD 2007-2022).

**Methods:**

As part of the CANWARD national surveillance study, sentinel hospitals across Canada submitted the first 10 clinically significant isolates recovered from blood cultures each month. Isolates underwent antimicrobial susceptibility testing using the broth microdilution method and susceptibility to antibiotics was determined whenever MICs and CLSI breakpoints were available. For *Streptococcus* and *Enterococcus,* where piperacillin-tazobactam breakpoints were not available, susceptibility was inferred from penicillin and ampicillin MICs as per EUCAST standards.

**Figure:**
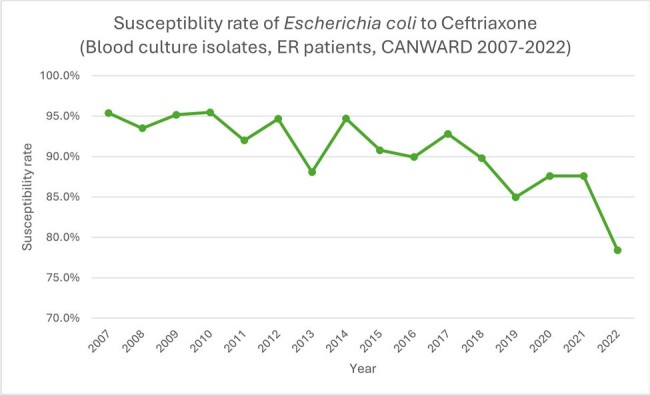

Rate of ceftriaxone susceptibility in blood culture Escherichia coli isolates (ER patients, CANWARD 2007-2022).

**Results:**

From 2007 to 2022, 9,178 blood culture isolates were collected from patients presenting to Canadian emergency rooms. The most common organisms isolated were:

1. Escherichia coli (31.7%)

2. Staphylococcus aureus (16.8%)

3. Klebsiella pneumoniae (7.8%)

4. Streptococcus pneumoniae (6.8%)

5. Streptococcus pyogenes (2.9%)

There has been a marked increase in the rate of ESBL production in E. coli (Figure 1). This is associated with a decrease in the susceptibility rate of E. coli to ceftriaxone (Figure 2).

Despite these changes, isolates from blood cultures of ER patients remain susceptible to piperacillin-tazobactam more than 90% of the time and remain susceptible to piperacillin-tazobactam plus vancomycin more than 95% of the time (Table 1).

**Figure:**
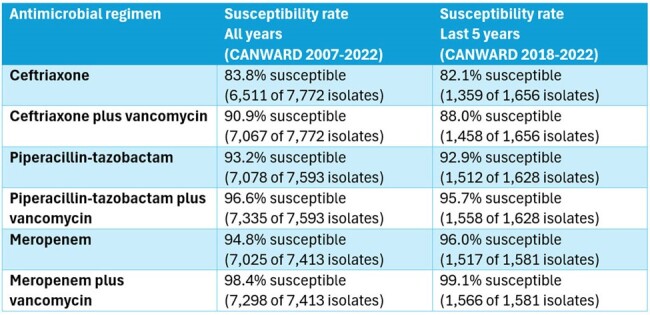

Susceptibility rates of blood culture isolates from ER patients to commonly prescribed empiric antimicrobial regimens.

**Conclusion:**

Isolates causing bloodstream infections in Canadian ER patients remain susceptible to piperacillin-tazobactam more than 90% of the time. The susceptibility rate to ceftriaxone is less than 85% and ceftriaxone should be used with caution in sepsis when the source of infection is unclear. Individual centers should perform their own assessments based on the most common pathogens causing bacteremia and their local susceptibility rates.

**Disclosures:**

**All Authors**: No reported disclosures

